# Case report: Near-fatal hypermagnesemia resulting from the use of Epsom salts in a patient with normal renal function

**DOI:** 10.3389/fmed.2024.1416956

**Published:** 2024-07-03

**Authors:** Gui-Fei Si, Yu-Xin Ge, Xiao-Pan Lv, Yu-Quan Li, Xue-Mei Chen, Xue-Min Yuan

**Affiliations:** ^1^School of Clinical Medicine, Shandong Second Medical University, Weifang, Shandong, China; ^2^Department of Gastroenterology, The People’s Hospital of Linyi, Linyi, Shandong, China

**Keywords:** hypermagnesemia, Epsom salts, magnesium sulfate, subtotal gastrectomy, normal renal function, peptic ulcer

## Abstract

Hypermagnesemia commonly occurs in patients with renal dysfunction. Diagnosing hypermagnesemia represents a challenge due to its rarity and the absence of routine monitoring of magnesium levels. Furthermore, the lack of awareness among clinicians regarding this uncommon condition frequently leads to delayed diagnoses. Few patients survive with a serum magnesium level exceeding 7 mmol/L. This article presents a case study of near-fatal hypermagnesemia resulting from the oral administration of Epsom salts in a patient with normal renal function. A 60-year-old female presented to the gastroenterology department on Oct. 6, 2023, with a 3-day history of black stools. She underwent subtotal gastrectomy in 2005 and has a stable history of nephrotic syndrome. To investigate the cause of her bleeding, electronic gastroscopy and colonoscopy were scheduled for Oct. 11, 2023. She experienced a sudden loss of consciousness 30 min after the ingestion of Epsom salts. The attending physician suspected a severe magnesium poisoning. She was promptly administered calcium gluconate, underwent tracheal intubation with ambu bag ventilation, and received early continuous renal replacement therapy (CRRT). Swift diagnosis and CRRT contributed to a reduction in her serum magnesium levels from an initial 8.71 mmol/L to 1.35 mmol/L, leading to a remarkable improvement in the toxic symptoms associated with hypermagnesemia. Subsequently, she was managed in the gastroenterology department, with gastroscopy revealing bleeding from the gastrointestinal anastomotic ulcer. Following conservative treatments including acid suppression, stomach protection, and hemostasis, her symptoms improved, and she was successfully discharged. This study aims to alert clinicians to the possibility of hypermagnesemia in individuals with normal renal function. Physicians should exercise caution when prescribing Epsom salts to patients with underlying gastrointestinal conditions. If necessary, alternative drug therapies may be considered to mitigate the risk of hypermagnesemia. Timely intervention is pivotal in averting life-threatening complications linked to hypermagnesemia.

## 1 Introduction

Magnesium salts play a pivotal role in various clinical applications. The pharmacological effects of different types of magnesium salts are specific to treating a wide range of diseases and symptoms. For instance, magnesium sulfate is predominantly utilized in obstetrics for the prevention of seizures in pre-eclampsia and recurrence in eclampsia (1, 2). It also acts as a neuroprotective agent in cases of preterm labor and in critically ill patients to manage severe hypomagnesemia (3–6). Intravenous magnesium sulfate stands as the mainstay treatment for torsades de pointes, while nebulized magnesium sulfate is advocated for severe asthma exacerbations (7–10). Magnesium sulfate is also utilized topically to treat sprains and hemorrhoids (11). Moreover, magnesium salts such as magnesium sulfate, magnesium citrate, magnesium hydroxide, and magnesium oxide are commonly used as physiological laxatives to relieve symptoms of constipation (6, 12, 13). Additionally, magnesium oxide, magnesium chloride, magnesium lactate, and magnesium carbonate are employed in the treatment of mild hypomagnesemia (14).

Hypermagnesemia is a rare and typically iatrogenic condition (15). It commonly arises in patients receiving excessive infusions for severe pre-eclampsia/eclampsia (16–18). Individuals with chronic kidney disease (CKD) or acute kidney injury face an elevated risk of hypermagnesemia (19). However, recent studies indicate that hypermagnesemia can also manifest in individuals with normal kidney function, particularly in elderly patients with specific gastrointestinal conditions (11, 20–26). Few patients survive with a serum magnesium level exceeding 7 mmol/L (27, 28). This article presents a unique case study of a patient who developed near-fatal hypermagnesemia, despite maintaining normal renal function and not surpassing the recommended upper limit of laxative salt intake.

## 2 Case presentation

A 60-year-old female presenting with a 3-day history of melena was hospitalized on Oct. 6, 2023. She underwent subtotal gastrectomy in 2005. She has a medical history of nephrotic syndrome, currently in a stable condition. Her renal function was within normal limits, with a creatinine level of 42.0 μmol/L, blood urea level of 6.6 mmol/L, and consistently undetectable urine protein levels. During examination, her heart rate was recorded at 109 beats per minute (bpm), blood pressure at 123/69 mmHg, respiratory rate at 18 bpm, and body temperature at 35.8°C. Physical assessment indicated slightly pale conjunctiva. Furthermore, an old longitudinal surgical scar, measuring approximately 15 cm in length, was observed in the midline of the abdominal wall. Laboratory analysis revealed the following: severe anemia (hemoglobin: 64.0 g/L, hematocrit: 20.10%), hypoproteinemia (serum albumin: 28.8 g/L), hypocalcemia (2.01 mmol/L), and hypomagnesemia (0.65 mmol/L). Liver function and other electrolyte parameters showed no abnormalities. A computed tomography (CT) scan of the entire abdomen showed postoperative gastric changes with a patent anastomotic site, low-density nodules in the quadrate hepatic lobe, a calcified lesion in the right hepatic lobe, and small kidney stones or calcified lesions bilaterally. Electrocardiography (ECG) displayed sinus rhythm (94 bpm), left ventricular high voltage (Rv5 + Sv1 = 4.17 mV), and abnormal T waves. Following symptomatic supportive interventions such as acid suppression, gastric protection, hemostasis, fluid resuscitation, and blood transfusion regimen, her condition stabilized with no recurrent bleeding. Considering her mild hypomagnesemia diagnosis, we refrained from initiating treatment with medications like magnesium oxide, magnesium chloride, magnesium lactate, or magnesium carbonate.

**Figure 1:**
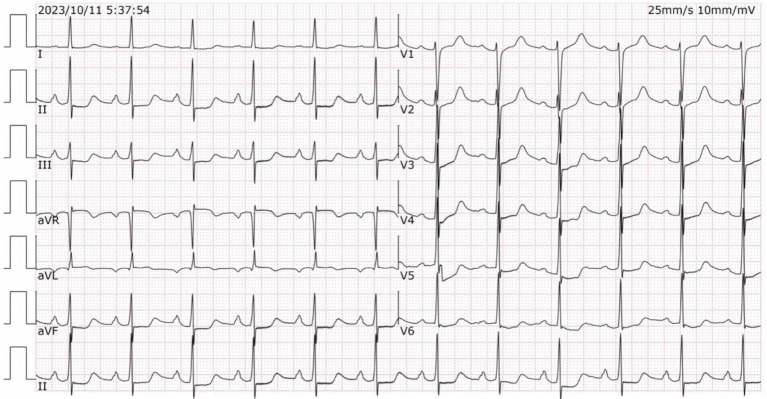
Patient’s electrocardiogram.

To investigate the root cause of the hemorrhage, she was scheduled for electronic gastroscopy and colonoscopy on Oct. 11, 2023. A solution made of 50 g of magnesium sulfate dissolved in 150 mL of warm water was administered for intestinal preparation. Approximately half an hour after ingestion, she suddenly lost consciousness. Electrocardiogram monitoring revealed a rapid elevation in heart rate (90–100 bpm), while oxygen saturation gradually declined to approximately 75%. She was uncooperative during the physical examination. The attending physician suspected magnesium poisoning as the preliminary diagnosis. She received immediate treatment with intravenous calcium gluconate (2 g) and a 500-mL intravenous infusion of 0.9% sodium chloride. A series of laboratory tests were promptly requested, with the results of the arterial blood gas analysis beginning to be available within about 20 min. The findings revealed a combination of respiratory acidosis, metabolic acidosis, and respiratory failure (pH: 7.225, pO2: 68.60 mmHg, pCO2: 57.1 mmHg, bicarbonate: 20.6 mmol/L). The remaining laboratory workup results were available within approximately 1 h after arrival. The laboratory analysis indicated mild anemia (hemoglobin: 100.0 g/L, hematocrit: 31.30%), hypermagnesemia (8.71 mmol/L), hypokalemia (2.75 mmol/L), and hypercalcemia (2.70 mmol/L). There were no abnormalities found in liver and renal function, myocardial enzyme spectrum, other electrolytes and random blood glucose (10.2 mmol/L) levels. The ECG (Figure 1) demonstrated normal sinus rhythm at a rate of 79 bpm, along with first-degree atrioventricular block (210 ms), ST segment depression, and prolonged QTc intervals (542 ms). The ECG did not exhibit any changes suggestive of acute myocardial infarction.

The intensive care unit (ICU) service was consulted for the treatment of hypermagnesemia. She was intubated and ventilated using a balloon, resulting in an increase in oxygen saturation to 94%. Subsequently, she was transferred to the ICU for further care. During the treatment, diprophylline injection was continuously infused to improve respiratory function and metaraminol bitartrate injection to maintain blood pressure. In addition, she was subsequently treated with 2 g of i.v. calcium gluconate to prevent arrhythmia, 20 mg of i.v. furosemide to promote renal excretion of magnesium.

**Figure 2:**
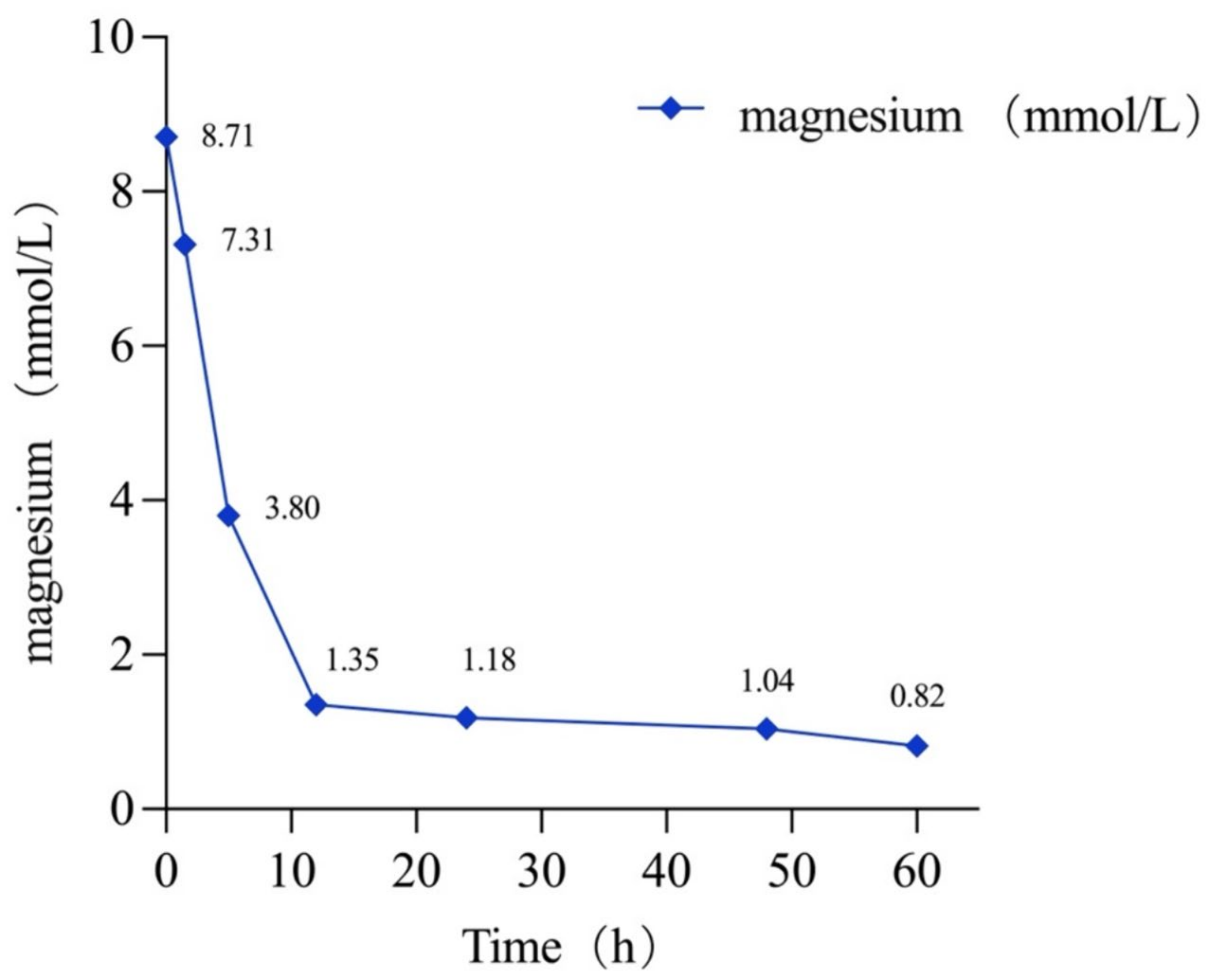
Changes in serum magnesium values over time.

However, her serum magnesium level remained >3.8 mmol/L in the ICU 5 h post-arrival. Consequently, she underwent emergent continuous renal replacement therapy (CRRT), resulting in a significant reduction in her serum magnesium level from >3.8 mmol/L to 1.35 mmol/L after a 7 h hemodialysis session. Subsequent monitoring showed further improvement, with her serum magnesium level decreasing to 1.18 mmol/L 12 h later. After being monitored for 36 h, she was successfully extubated the following afternoon. By day 3, her serum magnesium level had normalized, at 0.82 mmol/L (Figure 2). As her magnesium levels decreased, her level of consciousness gradually returned. Subsequently, she was managed in the gastroenterology department, with gastroscopy revealing bleeding from the gastrointestinal anastomotic ulcer. Following conservative treatments including acid suppression, stomach protection, and hemostasis, her symptoms improved, and she was successfully discharged.

## 3 Discussion

Magnesium is an indispensable mineral that serves as a vital cofactor in over 300 enzymatic reactions within the human body (15). It plays a critical role in DNA, RNA, and protein synthesis, as well as in adenosine triphosphate (ATP) metabolism (29). Furthermore, magnesium is essential for regulating a diverse array of physiological functions, including blood pressure, cardiac excitability, vasomotor tone, insulin metabolism, muscular contraction, nerve transmission, and neuromuscular conduction (30). The maintenance of magnesium homeostasis hinges on the coordinated interplay of renal excretion and reabsorption, gastrointestinal absorption and bones (23, 31).

The human body predominantly absorbs magnesium through the paracellular pathway in the jejunum and ileum, accounting for 80–90% of total absorption (32, 33). The majority of magnesium in the human body is stored in bones. Less than 1% of magnesium is present in extracellular fluid, with less than 0.3% found in serum (34, 35). The normal reference range for serum magnesium concentration is typically between 0.7–1.10 mmol/L (1.7–2.4 mg/dL) (30, 36). The kidneys play a crucial role in maintaining magnesium homeostasis (15). Under normal magnesium levels, the kidneys filter a daily amount of magnesium ranging from 2000 to 2,400 mg, with approximately 96% of this being reabsorbed through paracellular mechanisms, primarily in the thick ascending limb of the loop of Henle. Only 3–5% of magnesium is excreted in the urine (37). In cases of hypermagnesemia, healthy kidneys can completely inhibit magnesium reabsorption from the loop of Henle, leading to potent magnesium excretion by the kidneys (38). Consequently, in individuals with normal renal function, hypermagnesemia is exceedingly rare.

Hypermagnesemia is a potentially life-threatening complication often associated with renal insufficiency (GFR < 30 mL/min), intravenous magnesium sulfate administration, excessive oral intake of magnesium-containing medications, high-dose magnesium sulfate enemas, and the use of antacids. In addition to these common causes, less frequent contributors include conditions such as hypothyroidism, hyperparathyroidism, adrenal insufficiency, rhabdomyolysis, and tumor lysis syndrome (39, 40). Additionally, constipation and the administration of specific medications, such as vitamin D, anticholinergics, and opiates, may elevate the risk of hypermagnesemia in individuals with normal renal function (21, 23, 39). A retrospective review of the patient’s medical history, conducted after symptom resolution, did not reveal any of the risk factors for hypermagnesemia. Patients with hypermagnesemia may experience a progressive inhibition of neuromuscular, cardiovascular, and respiratory system functions (41). The clinical manifestations of hypermagnesemia are dependent on serum magnesium concentrations. Symptoms such as fatigue, nausea, vomiting, and decreased tendon reflexes typically occur when serum levels reach 5 mg/dL (2.06 mmol/L). Prolongation of the Q-T intervals, loss of deep tendon reflexes, and somnolence are observed at serum levels between 5–12 mg/dL (2.06–4.94 mmol/L). Severe symptoms including coma, cardiac arrest, respiratory failure, and death can occur at serum levels exceeding 10–15 mg/dL (4.12–6.17 mmol/L) (22, 42–44).

The primary focus of initial treatment for hypermagnesemia involves continuous cardiac monitoring and airway management (25). In cases of life-threatening complications, the administration of intravenous calcium salts serves as emergency therapy. Calcium not only reverses cardiac dysrhythmias and respiratory depression but also alleviates hypocalcemia resulting from hypermagnesemia (40). It is worth noting that the patient displays hypermagnesemia and hypercalcemia, a presentation significantly divergent from previous research findings. Our speculation is that this divergence could be attributed to the on-duty physician’s initial administration of calcium gluconate therapy relying on clinical experience, contributing to subsequent alterations in laboratory test results. This underscores the importance of continuous monitoring of serum magnesium and calcium levels throughout the entire therapeutic course. Moreover, the administration of intravenous normal saline is crucial in the management and prophylaxis of hypermagnesemia in high-risk patients, particularly individuals with intestinal dysfunction who use magnesium as a laxative. The sodium chloride content in normal saline is advantageous in preserving appropriate urine output and facilitating the renal excretion of excess magnesium (26). Additionally, intravenous normal saline can aid in averting the buildup of magnesium in the blood, diminishing the serum magnesium levels and thereby decreasing the likelihood of hypermagnesemia in such patients. Furosemide may be administered to enhance the renal excretion of magnesium. For individuals with renal impairment, prompt hemodialysis should be initiated (21). In this study, the decision to proceed with hemodialysis was made due to the patient’s escalating serum magnesium levels and critical clinical state despite normal renal function. Following treatment, her symptoms of poisoning have shown significant improvement.

Following an in-depth literature review, we discovered limited case reports of patients who survived severe hypermagnesemia (27, 28, 45). In this study, the patient with normal renal function experienced near-fatal hypermagnesemia while using standard doses of magnesium sulfate for intestinal preparation. We considered that several possible factors could account for the occurrence of hypermagnesemia in this case. Initially, hydration status plays a pivotal role in regulating the levels of ionized electrolytes in the serum and their impact on organ function. Dehydration secondary to bowel preparation could potentially elevate the likelihood of relative hypovolemia, thereby predisposing the individual to hypermagnesemia (27, 46). Moreover, the patient’s history of subtotal gastrectomy may affect hydration status by influencing gastric emptying and the elimination of magnesium sulfate. Typically, it takes 4–6 h for the stomach to fully empty medication. Once magnesium sulfate enters the intestinal cavity, minimal absorption occurs, with most of it remaining in the intestines. The presence of retained magnesium sulfate elevates osmotic pressure in the intestines, leading to water retention and the absorption of water from surrounding tissues into the intestinal cavity. This increased volume stimulates the intestinal wall, enhancing peristalsis, facilitating fecal excretion, and completing the intestinal preparation process. Following a subtotal gastrectomy, the pylorus loses its normal function, resulting in a notable delay in gastric emptying. Undiluted magnesium sulfate swiftly moves from the stomach into the intestinal cavity. This rapid influx causes the jejunum to expand quickly, leading to a substantial transfer of extracellular fluid into the intestinal cavity, ultimately culminating in dehydration and a decrease in circulating blood volume. This further aggravates the imbalance in hydration status, making patients more susceptible to developing hypermagnesemia. Furthermore, the patient experienced gastrointestinal bleeding, with subsequent gastroscopy revealing bleeding from the gastrointestinal anastomotic ulcer. Given the presence of active gastric ulcer disease, which can increase magnesium absorption, the likelihood of an elevated magnesium concentration is significantly higher (20). Additionally, the elderly female patient with sluggish intestinal peristalsis faces an increased risk. In cases of hypermagnesemia, interference and blockage of myenteric neuronal function can disrupt the excitation-contraction coupling of smooth muscle cells, further exacerbating intestinal peristalsis issues (47). This disturbance allows for the continuous uptake of ingested magnesium. Magnesium remains in the intestinal cavity for an extended period and is continuously absorbed, resulting in persistent hypermagnesemia.

The case of the patient described here draws parallels to a 2020 report by Philip et al. on hypermagnesemia resulting from an Epsom salts overdose (44). In both instances, elderly patients displayed symptoms of magnesium toxicity, such as hypotension, shock, and respiratory failure, post magnesium sulfate administration. Treatment for both cases involved similar interventions, including calcium administration to counteract myocardial toxicity, respiratory support via endotracheal intubation, fluid support, and furosemide to enhance magnesium excretion. Notably, both patients exhibited positive outcomes with prompt hypermagnesemia correction through timely CRRT. However, significant differences exist between the two cases. Compared to Philip et al.’s report, the Epsom salt quantity was lower, onset was swifter, serum magnesium levels were higher, and symptoms were more pronounced in the current patient. The condition’s etiology may be influenced by factors like gender, prior gastric surgery, and active bleeding peptic ulcers, impacting magnesium metabolism and excretion, potentially leading to more severe symptoms. A comparative analysis of these cases offers a deeper understanding of hypermagnesemia’s etiology, clinical signs, and treatment strategies, aiding in more effective management of similar clinical scenarios.

## 4 Conclusion

This study has provided us with a valuable clinical insight. Hypermagnesemia can occur in patients who have undergone subtotal gastrectomy or peptic ulcer, even when the magnesium sulfate dosage is within the recommended limits and renal function is normal. It is imperative for physicians to exercise caution and monitor vital signs and serum magnesium levels when prescribing or increasing magnesium sulfate for such patients to avert symptomatic hypermagnesemia. Physicians are encouraged to recommend alternative laxatives, such as sodium picosulfate or polyethylene glycol electrolyte powder, as viable options for these patients. Early intervention is paramount in preventing life-threatening complications associated with hypermagnesemia. Upon diagnosing hypermagnesemia, prompt supportive measures should be initiated, including intravenous calcium supplementation, loop diuretics, fluid replacement, and emergency hemodialysis.

## Data availability statement

The original contributions presented in the study are included in the article/supplementary material, further inquiries can be directed to the corresponding author.

## Ethics statement

The studies involving humans were approved by Science and Technology Ethics Committee Linyi People’s Hospital. The studies were conducted in accordance with the local legislation and institutional requirements. The participants provided their written informed consent to participate in this study. Written informed consent was obtained from the individual(s) for the publication of any potentially identifiable images or data included in this article. Written informed consent was obtained from the participant/patient(s) for the publication of this case report.

## Author contributions

G-FS: Conceptualization, Data curation, Methodology, Software, Visualization, Writing – original draft, Writing – review & editing. Y-XG: Investigation, Writing – review & editing. X-PL: Writing – review & editing. Y-QL: Writing – review & editing. X-MC: Data curation, Writing – review & editing. X-MY: Funding acquisition, Visualization, Writing – review & editing.
